# tRF-003634 alleviates adriamycin-induced podocyte injury by reducing the stability of TLR4 mRNA

**DOI:** 10.1371/journal.pone.0293043

**Published:** 2023-10-19

**Authors:** Xiaoqing Gao, Yunyang Qiao, Shanwen Li, Huimin Shi, Gaoting Qu, Jialing Ji, Weihua Gan, Aiqing Zhang

**Affiliations:** 1 Department of Pediatric Nephrology, The Second Affiliated Hospital of Nanjing Medical University, Nanjing, Jiangsu, China; 2 Department of Pediatrics, The Fourth Affiliated Hospital of Nanjing Medical University, Nanjing, Jiangsu, China; University of Utah School of Medicine, UNITED STATES

## Abstract

Podocyte injury plays a key role in the production of proteinuria and is closely related to the progression of chronic kidney disease (CKD). Alleviating podocyte injury is beneficial to prevent the occurrence and development of CKD. tRNA-derived RNA fragments (tRFs) are associated with podocytes injury processes such as protein binding, cell adhesion, synapses, the actin cytoskeleton. Our previous data showed that tRF-003634 tightly correlated with podocyte injury, while its effect remains unclear. This study aimed to investigate the role of tRF-003634 in podocyte injury and the potential mechanisms. The expression level of tRF-003634, nephrin, podocin and tRF-003634 targeted toll-like receptor 4 (TLR4) in podocytes and kidney tissues were examined by quantitative real-time PCR (qRT-PCR), western blot and immunohistochemistry. The biochemical indices were monitored and renal pathological changes were assessed by hematoxylin and eosin PAS staining. Furthermore, potential target genes of tRF-003634 were screened using high-throughput mRNA sequencing, and then confirmed by RNA pulse-chase analysis. The results showed that tRF-003634 was downregulated in adriamycin (Adr)-induced podocyte injury. Overexpression of tRF-003634 increased the expression of nephrin and podocin in vivo and in vitro and alleviated podocyte injury. Meanwhile, overexpression of tRF-003634 alleviated proteinuria and renal pathological damage. In addition, high-throughput sequencing after overexpression of tRF-003634 showed that TLR4 might be a downstream target gene. tRF-003634 can alleviate podocyte injury by reducing the stability of TLR4 mRNA, possibly by competing with TLR4 mRNA to bind to YTH domain-containing protein 1 (YTHDC1). In conclusion, tRF-003634 was underexpressed in Adr-induced podocyte injury, and its overexpression alleviated podocyte injury in vitro and in vivo by reducing the stability of TLR4 mRNA.

## Introduction

Chronic kidney disease (CKD) has gradually become a major global public health concern. The prevalence of CKD in children and young people has shown a continuously increasing trend [1]. As the disease progresses, CKD eventually develops into end-stage renal disease, and its poor prognosis and high treatment costs pose huge challenges for patients and public health systems [2]. However, the onset of CKD is relatively insidious and lacks specific clinical manifestations in its early stages. It is often not diagnosed until the late stages of the disease when the opportunity to protect the glomerular filtration rate and reduce cardiovascular complications and mortality has been lost. Therefore, early diagnosis and intervention of CKD have important clinical value and practical significance.

Among the current treatment strategies for CKD, the control of proteinuria has been widely recognized, and its mechanism of action has been further explored [3]. Proteinuria is an important sign of renal injury and an independent risk factor for progression of CKD [4]. Podocytes are key cells that affect CKD occurrence and development [5]. They are terminally differentiated visceral glomerular epithelial cells located in the outermost layer of the glomerular basement membrane (GBM). Podocytes have many functions, such as synthesizing GBM components and maintaining the metabolic balance of GBM, resisting intra-glomerular pressure, regulating the filtration coefficient, and maintaining the stability of the glomerular capillary network. Podocytes maintain the normal structure and function of the filtration barrier [6]. Destruction of this barrier is the main cause of proteinuria. Podocyte injury plays a pivotal role in proteinuria and is closely linked to the progression of CKD [7]. Podocyte injury involves changes in the proteins of the slit membrane and cytoskeleton, surface charge of podocytes, interaction between podocytes and the GBM, and apoptosis. Oxidative stress, mitochondrial dysfunction, disturbances in the insulin signaling pathway, inflammation, and autophagy can all contribute to podocyte injury. Decoding the mechanism of podocyte injury may provide more avenues for preventing and treating CKD.

Small non-coding RNAs belong to the non-coding RNA family and play important roles in many pathological processes. They include newly-discovered tRNA-derived fragments (tRFs), which play a significant role in biological progression and disease occurrence; their mechanism of action is similar to that of miRNAs [8]. tRFs are produced by specific nucleases such as Dicer and angiogenin (ANG) at specific sites of tRNA and are approximately 16–35 nucleotides in length. They regulate the expression of protein-coding genes at the post-transcriptional or translational level by combining with mRNA 3′-UTR, 5′-UTR, or RNA binding protein (RBP), and then participate in a series of important life activities, for instance, cell proliferation, apoptosis, and differentiation [9, 10]. Abnormal expression of tRFs plays a significant regulatory role in the occurrence and progression of neuronal degeneration, tumors, infection, immune regulation, and kidney disease [11, 12]. Sequencing analysis of exosomes extracted from the urine of CKD patients found many differentially expressed tRFs, among which tRFVal and tRFLeu derived from the 5′ end of mature tRNA had significantly reduced expression. In addition, after culturing renal tubular epithelial cells in vitro and inducing injury, their expression was reduced, suggesting their potential value as an early diagnosis and therapeutic target for CKD [12].

In our previous study, high-throughput sequencing detected the expression levels of tRFs in differentiated and undifferentiated podocytes, and their target genes were analyzed. The results showed that these tRFs are involved in transcription, angiogenesis, cell adhesion, and the PI3K-Akt, Ras, Wnt, and MAPK signaling pathways that play major roles in the occurrence and development of CKD. tRFs are involved in podocyte injury, which plays an important role in proteinuria, suggesting that tRFs may have an important regulatory function in proteinuria [13].

Adriamycin (Adr) was used to induce podocyte injury to investigate the potential function of tRFs. Subsequently, using high-throughput sequencing the tRFs differentially expressed in the Adr-treated and normal cell groups were screened. Biological analysis methods found that they may be associated with podocyte injury through the PI3K-Akt, Wnt, and Ras signaling pathways [14]. This study investigated tRF-003634, which may be associated with podocyte injury. The role of tRF-003634 in Adr-induced podocyte injury was observed in animal and cell experiments, and its possible mechanism of action was discussed.

## Materials and methods

### Cell lines and culture conditions

The mouse podocyte cell line (MPC5) were donated by the Nephrology Research Center of the Second Affiliated Hospital of Nanjing Medical University. Undifferentiated podocytes were initially cultured in RPMI-1640 medium (Hyclone Laboratories Inc., USA) containing 10% fetal bovine serum (Gibco, USA), 100 U/mL penicillin, 100 U/mL streptomycin, and 10 U/mL interferon-γ (PeproTech, USA). Cells were incubated at 33°C with 5% CO_2_. To induce differentiation, podocytes were transferred to type I collagen-coated culture flasks and cultured at 37°C in medium without interferon-γ for 14 days. Differentiation was then confirmed using podocyte markers. Differentiated podocytes were used in subsequent experiments.

### Cell transfection

Differentiated podocytes were plated in six-well plates and incubated in serum-free RPMI-1640 medium for 24 h, and all cells synchronously entered quiescence. The tRF-003634 mimetics (5’-TAGTGGTTAGTACTCTG-3’, RiboBio, China) and tRF-003634 negative control (NC) (5’-CUGUGUUGAAUUACGGU-3’, RiboBio, China) were synthesized. The groups were as follows: (1) Control (2) Adr group (3) tRF-003634 NC (4) tRF-003634 mimetic (5) Adr+tRF-003634 NC (6) Adr+tRF-003634 mimetic. Lipofectamine 2000 (Invitrogen, Thermo Fisher Scientific Inc., USA) was used for transfection according to the instructions. The liposomes, tRF-003634 mimetic, and negative control were first diluted with serum-free medium. Subsequently, equal volumes of liposomes and tRF-003634 mimetic or NC were mixed gently and left at room temperature for 5 min before being added to the cells. The concentration of both the tRF-003634 mimetic and NC was 50 nM and both were incubated for 6 h. And then, the medium was replaced with complete medium, and Adr (0.1 μg/mL) was placed into the Adr group, Adr+tRF-003634 NC group, and Adr+tRF-003634 mimetic group for 24 h. Total RNA or protein was finally extracted for detection.

### Animal models and specimen collection

The mice used in this study were provided by Nanjing Qinglongshan Animal Breeding Company. Twenty wild-type SPF grade BALB/c mice (male, 5–6 weeks old, 23–25 g) were randomly selected. The mice were subjected to adaptive rearing before the experiment, controlled room temperature (23 ± 2°C) and humidity (55 ± 5%), a 12 h light/dark cycle, and specific pathogen-free conditions for 7 days, followed by experimental use. The animal experiment was performed in accordance with the ARRIVE guidelines and the National Institutes of Health Guide for the Care and Use of Laboratory Animals.

Twenty mice were randomly divided into four groups and housed in separate cages. tRF-003634 agomir (5’-TAGTGGTTAGTACTCTG-3’, RiboBio) and tRF-003634 NC (5’-CUGUGUUGAAUUACGGU-3’, RiboBio) working solutions were prepared according to the manufacturer’s instructions, and the mice were treated according to reference and preliminary experimental results [15]. An Adr nephropathy mouse model was induced by tail vein injection of Adr. tRF-003634 agomir overexpressed tRF-003634, while tRF-003634 NC were used as the negative controls. The groups were divided and treated as follows (n = 5 per group): (1) Control group: 0.9% NaCl was injected into the tail vein. (2) Adr group: one-time tail vein injection of Adr (10 mg/kg). (3) Adr+tRF-003634 NC group: one-time tail vein injection of Adr (10 mg/kg), tail vein injection of 50 nM tRF empty reagent on days 4, 8, and 12; continued for 14 days. (4) Adr+tRF-003634 agomir group: one-time tail vein injection of Adr (10 mg/kg), tail vein injection of 50 nM tRF agomir reagent on days 4, 8, and 12; continued for 14 days. On the 13th day after Adr injection, the mice were placed in metabolic cages with normal food and water, and 24 h urine was collected. The urine volume was measured, centrifuged at 4°C, 3000 rpm for 3 min, and the supernatant was collected. All mice were euthanized by intraperitoneal injection of barbital sodium (100mg/Kg) on the 14th day. Every effort was made to minimize the suffering of sacrificed animals in experiments, which were performed strictly in accordance with the Guidelines for the Care and Use of Laboratory Animals. All experiments were approved by the Institutional Animal Care and Use Committee of Nanjing Medical University (No. IACUC-2204009). The blood was collected in an anticoagulation tube, centrifuged at 4°C, 3000 rpm for 5 min, and the supernatant was collected. Levels of 24-h urine protein, serum creatinine (SCr), and blood urea nitrogen (BUN) were detected separately according to the manufacturer’s instructions (Jiancheng Bioengineering Inst., China). Renal pathological changes were observed by hematoxylin and eosin PAS staining (Solarbio, China). The expression level of nephrin and podocin was detected using quantitive real-time PCR (qRT-PCR), western blot, and immunohistochemistry.

### RNA extraction and qRT-PCR

Total RNA from the cells or kidney tissues was extracted using a TRIzol kit (Thermo Fisher, USA), according to the manufacturer’s protocol. rtStar^TM^ tRF&tiRNA pretreatment kits (Arraystar, USA) were used to remove the terminal and internal modifications from the total RNA to improve the efficiency of qRT-PCR. RNA concentration and purity were quantified using a NanoDrop spectrophotometer. Subsequently, qRT-PCR for tRF-003634 was performed. According to the manufacturer’s instructions, the Bulge-Loop™ miRNA qRT-PCR Starter Kit (RiboBio, China) was used to reverse-transcribe RNA to cDNA and perform PCR amplification. The amplification procedures were as follows: initial denaturation at 95°C for 10 min; denaturation at 95°C for 15 s; annealing at 60°C for 1 s; and extension at 72°C for 30 s. The expression level of tRF-003634 was normalized against that of U6 (RT primer, forward primer, and reverse primer of U6 and tRF-003634 were purchased from RiboBio). In addition, qRT-PCR for mRNA was performed as follows: total RNA was reversed into cDNA according to the instructions of HiScript III RT SuperMix for qPCR (+gDNA wiper) (Vazyme, China). The target gene was amplified by PCR according to the instructions of ChamQ Universal SYBR qPCR Master Mix (Vazyme, China). The thermocycling conditions were as follows: initial denaturation at 95°C for 30 s; denaturation at 95°C for 5 s; and annealing and extension at 60°C for 30 s. The expression levels of the target genes were detected using GAPDH as an internal reference. The primer sequences are shown in Table 1.

**Table 1:** Sequences of PCR primers.

	Forward and reverse primers
GAPDH-F	5′- TGGATTTGGACGCATTGGTC -3′
GAPDH-R	5′- TTTGCACTGGTACGTGTTGAT -3′
Nephrin-F	5′- ATGGGAGCTAAGGAAGCCACA -3′
Nephrin-R	5′- GATGGAGAGGATTACGCTGGG -3′
Podocin-F	5′- GCATCAAGCCCTCTGGATTAG -3′
Podocin-R	5′- AGACGGAGATCAACCTTGTGATA -3′

### Western blot analysis

Total protein of cells or kidney tissue was extracted with RIPA lysis buffer (ThermoFisher, USA), and protein concentrations were detected using a bicinchoninic acid (BCA) protein assay kit (KeyGen, China). Equal amount of protein (20 μg) in each group was separated by SDS-PAGE and then transferred to a PVDF membrane (Merck Millipore, USA). The membranes were blocked with 5% bovine serum albumin for 1 h at room temperature. After blocking, the PVDF membrane was soaked in primary antibody for 1 h at room temperature and then incubated at 4°C overnight. After washing with TBST for three times, the membrane was incubated with goat anti-mouse IgG HRP-conjugated secondary antibody (1:5000, S0002, Affinity, USA) at room temperature for 1 h. The membrane was washed again and then added ECL substrates (ThermoFisher, USA); band densities were quantified using ImageJ (NIH, USA). The expression levels of nephrin and podocin in podocytes or mouse kidney tissues in each group were detected. The primary antibodies used were: GAPDH (1:1000, 2118, Cell Signaling, USA), anti-nephrin (1:1000, ab58968, Abcam, UK), anti-podocin (1:1000, ab50339, Abcam, UK), anti-toll-like receptor 4 (TLR4, 1:1000, AF7017, Affinity, USA), anti-IL-6 (1:1000, DF6087, Affinity, USA), and anti-TNF-α (1:500, AF7014, Affinity, USA).

### High-throughput sequencing and target gene prediction

Differentiated podocytes were subjected to high-throughput sequencing after the Adr and Adr+tRF-003634 mimetic groups were treated for 24 h. The differentially expressed target genes associated with podocyte injury were screened; those with significantly low expression in the Adr+tRF-003634 mimetic group were selected. Target gene expression was verified using qRT-PCR. The expression of target genes, IL-6 and TNF-α, in the podocytes of the control group, Adr group, and Adr+tRF-003634 mimetic group was detected by qRT-PCR and western blot. The primer sequences were designed and synthesized by Shanghai Generay Biotechnology Co. Ltd and shown in Table 2.

**Table 2:** Sequences of PCR primers.

	Forward and reverse primers
TLR4-F	5′- TGTTCTTCTCCTGCCTGACA -3’
TLR4-R	5′- TGTCATCAGGGACTTTGCTG -3’
PHB2-F	5′- ACCGTGGAAGGCGGTCATA -3’
PHB2-R	5′- GGTCTGGCCCGAATGTCATAG -3’
IL-6-F	5′- CTGCAAGAGACTTCCATCCAG -3’
IL-6-R	5′- AGTGGTATAGACAGGTCTGTTGG -3’
TNF-α-F	5′- CCTTATCTACTCCCAGGTTCTC -3’
TNF-α-R	5′- GAGGCTGACTTTCTCCTGGTATG -3’
YTHDC1-F	5′- CATTTCAGAACGCACCAACAA -3’
YTHDC1-R	5′- GCAGAGCTGGGTTCCACAATC -3’

Using bioinformatic methods and the RNA-binding protein prediction website (http://rbpdb.ccbr.utoronto.ca/), the RBPs shared by tRF-003634 and the target genes were screened, and their binding sites were predicted.

### Effect of RBP on target gene mRNA stability

The differentiated podocytes were plated in 12-well plates, and RBP was knocked down using small interfering RNA (siRNA) technology. Using the RBP NC group as a negative control, the differentiated podocytes were divided into the RBP NC and RBP siRNA group (75 nM) and transfected using Lipofectamine 2000 (Invitrogen, Thermo Fisher Scientific Inc., USA). The cells were incubated in a 37°C incubator for 6 h and then the medium was replaced. The interference efficiency was verified by qRT-PCR. At various time points, the cells were treated with actinomycin D (5 μg/mL) to terminate mRNA synthesis. Cells were harvested at different time points (0, 1, 2, 4, 6, and 8 h), and RNA was extracted. Target gene mRNA expression was detected by qRT-PCR. Curves were drawn to analyze the effect of RBP on target gene mRNA stability.

### Effect of tRF-003634 on target gene mRNA stability

tRF-003634 was overexpressed in differentiated podocytes, and tRF-003634 NC was used as a negative control. Differentiated podocytes were divided into tRF-003634 NC and tRF-003634 mimetic groups, with six replicate wells in each group corresponding to six time points. At various time points, the cells were treated with actinomycin D (5 μg/mL) to terminate mRNA synthesis. Cells were harvested at different time points (0, 1, 2, 4, 6, and 8 h), and then RNA was extracted. Target gene mRNA expression was detected by qRT-PCR. Curves were drawn to analyze the effect of tRF-003634 on target gene mRNA stability.

### Statistical analysis

All results based on three biological replicates. Statistics and analysis of data were performed using GraphPad Prism 8.0 (GraphPad Software, Inc., USA). Measurement data were expressed as mean ± standard deviation. Student’s *t*-test was used for comparison between two groups. Comparisons among multiple groups were performed using one-way analysis of variance (ANOVA). P<0.05 indicated that the difference was considered statistically significant.

## Results

### Expression of tRF-003634 was downregulated in Adr-induced podocyte injury

**Fig 1:**
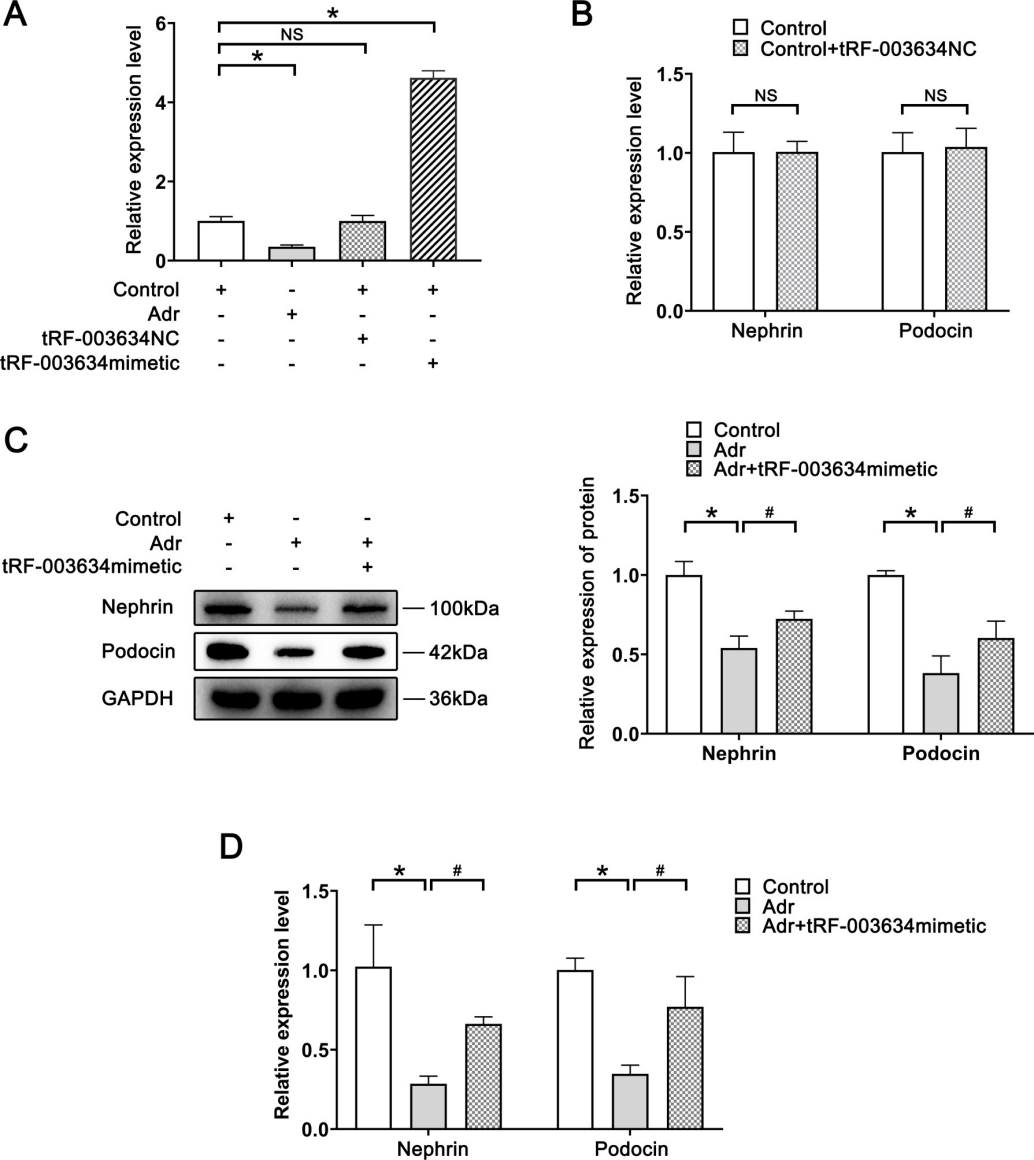
tRF-003634 overexpression attenuates podocyte injury in vitro. (A) Expression levels of tRF-003634 in each group were determined by qRT-PCR. (B) Expression levels of nephrin and podocin in control group and tRF-003634 NC group were detected by qRT-PCR. (C) Expression levels of nephrin and podocin in Adr group and tRF-003634 mimetic group were verified by western blot. (D) Expression levels of nephrin and podocin in Adr group and tRF-003634 mimetic group were verified by qRT-PCR. n = 3, *P<0.05 vs. the control group; #P<0.05 vs. the Adr group. NS, non-significant; tRF-003634 NC, tRF-003634 negative control.

The expression level of tRF-003634 was detected using qRT-PCR. The expression level of tRF-003634, compared with the control group, was significantly decreased in the Adr group (P<0.05), suggesting that tRF-003634 expression was downregulated in Adr-induced podocyte injury. Additionally, compared with the control group, the expression level of tRF-003634 in the tRF-003634 mimetic group was significantly increased (P<0.05), but not obviously different in the tRF-003634 NC group, indicating that overexpression of tRF-003634 was successful, and the empty load of tRF-003634 had no obvious interference effect (Fig 1A).

### tRF-003634 overexpression attenuated podocyte injury both in vitro and in vivo

To clarify the role of tRF-003634 in Adr-induced podocyte injury, we detected changes in the expression level of nephrin and podocin in podocytes and kidney tissues by qRT-PCR, western blot, and immunohistochemical methods. In addition, the biochemical indices and renal damage of mice with Adr nephropathy were monitored to observe podocyte damage.

Podocytes were transfected to overexpress tRF-003634, and tRF-003634 NC was used as a negative control. qRT-PCR showed that the expression levels of nephrin and podocin in the tRF-003634 NC group did not change significantly compared to the control group, suggesting that tRF emptying had no significant effect on podocyte injury (Fig 1B). Western blot and qRT-PCR proved that compared with the control group, the expression of nephrin and podocin in the Adr group was significantly decreased (P<0.05). Compared to the Adr group, the expression of nephrin and podocin in the Adr+tRF-003634 mimetic group was increased remarkably (P<0.05) (Fig 1C and 1D).

**Fig 2:**
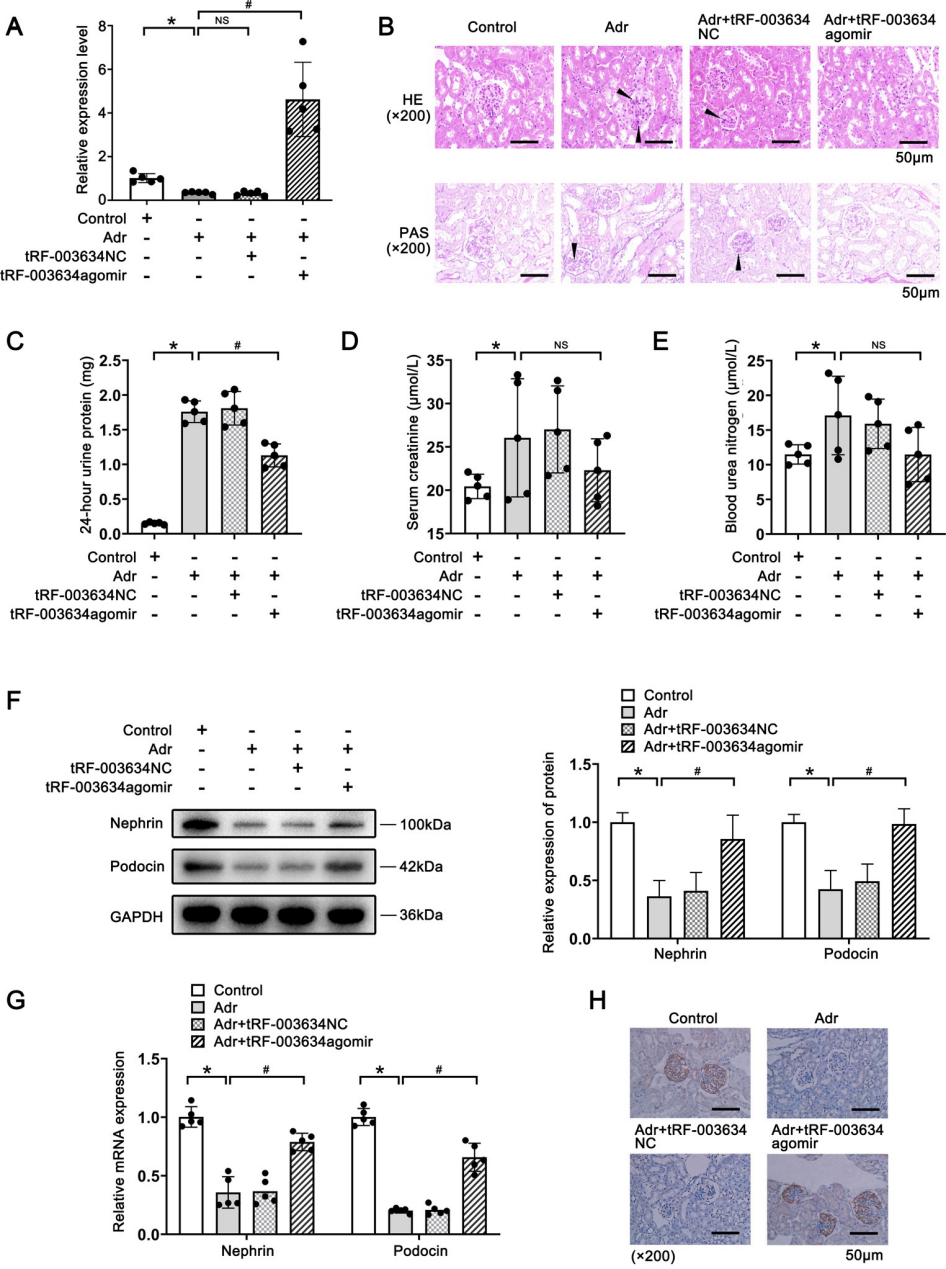
tRF-003634 overexpression attenuates podocyte injury in vivo. (A) Expression levels of tRF-003634 in each group were determined by qRT-PCR. (B) Morphological changes of mouse kidney (200x magnification; Scale Bar, 50μm). (C) The 24-h urine protein quantification in each group. (D) The serum creatinine levels in each group. (E) The blood urea nitrogen levels in each group. (F) Expression levels of nephrin and podocin in kidney tissue were detected by western blot. (G) Expression levels of nephrin and podocin in kidney tissue were detected by qRT-PCR. (H) Immunohistochemical verification of nephrin expression (200x magnification; Scale Bar, 50μm). n = 5, *P<0.05 vs. the control group; #P<0.05 vs. the Adr group. Black arrows: the increased glomerular mesangial matrix and balloon adhesion. NS, non-significant; tRF-003634 NC, tRF-003634 negative control.

To explore the role of RF-003634 in vivo, Adr, tRF-003634 agomir, and tRF-003634 NC were injected into the tail veins of different groups of BALB/c mice (n = 5 per group). qRT-PCR showed that, compared with the control group, the expression level of tRF-003634 was significantly decreased in the Adr group. Compared with the Adr group, the expression level of tRF-003634 was significantly increased in the Adr+tRF-003634 agomir group (P<0.05), while it did not change significantly in the Adr+tRF-003634 NC group, indicating that overexpression of tRF-003634 was successful (Fig 2A). Under a light microscope, the morphological changes in the kidneys were as follows: by HE and PAS staining, the kidney tissue of the mice in the Adr group showed increased glomerular mesangial matrix and number of cells, balloon adhesion, and renal tubular damage. In contrast, pathological kidney damage was alleviated in the Adr+tRF-003634 agomir group (Fig 2B). The 24-h urine protein quantification in the Adr group was significantly higher than that in the control group (P<0.05). Compared with the Adr group, the 24-h urine protein level of mice in the Adr+tRF-003634 agomir group was significantly lower (P<0.05), while the Adr+tRF-003634 NC group showed no significant difference (Fig 2C). Levels of SCr and BUN were significantly increased in the Adr group(P<0.05), compared with the control group. However, there was no obvious difference between the Adr and Adr+tRF-003634 agomir groups (Fig 2D and 2E). The expression levels of nephrin and podocin were detected to observe injury in mouse podocytes. Western blot and qRT-PCR showed that compared with the control group, the protein and mRNA expression levels of nephrin and podocin in the Adr group were significantly decreased (P<0.05). Compared with the Adr group, the protein and mRNA expression levels of nephrin and podocin in the Adr+tRF-003634 agomir group were significantly increased (P<0.05), while the expression levels of nephrin and podocin in the Adr+tRF-003634 NC group were not significantly different (Fig 2F and 2G). Immunohistochemical results indicated that compared to the control group, nephrin levels decreased in the Adr group. Compared with the Adr group, nephrin expression increased in the Adr+tRF-003634 agomir group, but it did not change significantly in the Adr+tRF-003634 NC group (Fig 2H). Overall, tRF-003634 overexpression attenuates podocyte injury.

### TLR4 was the downstream target gene regulated by tRF-003634

**Fig 3:**
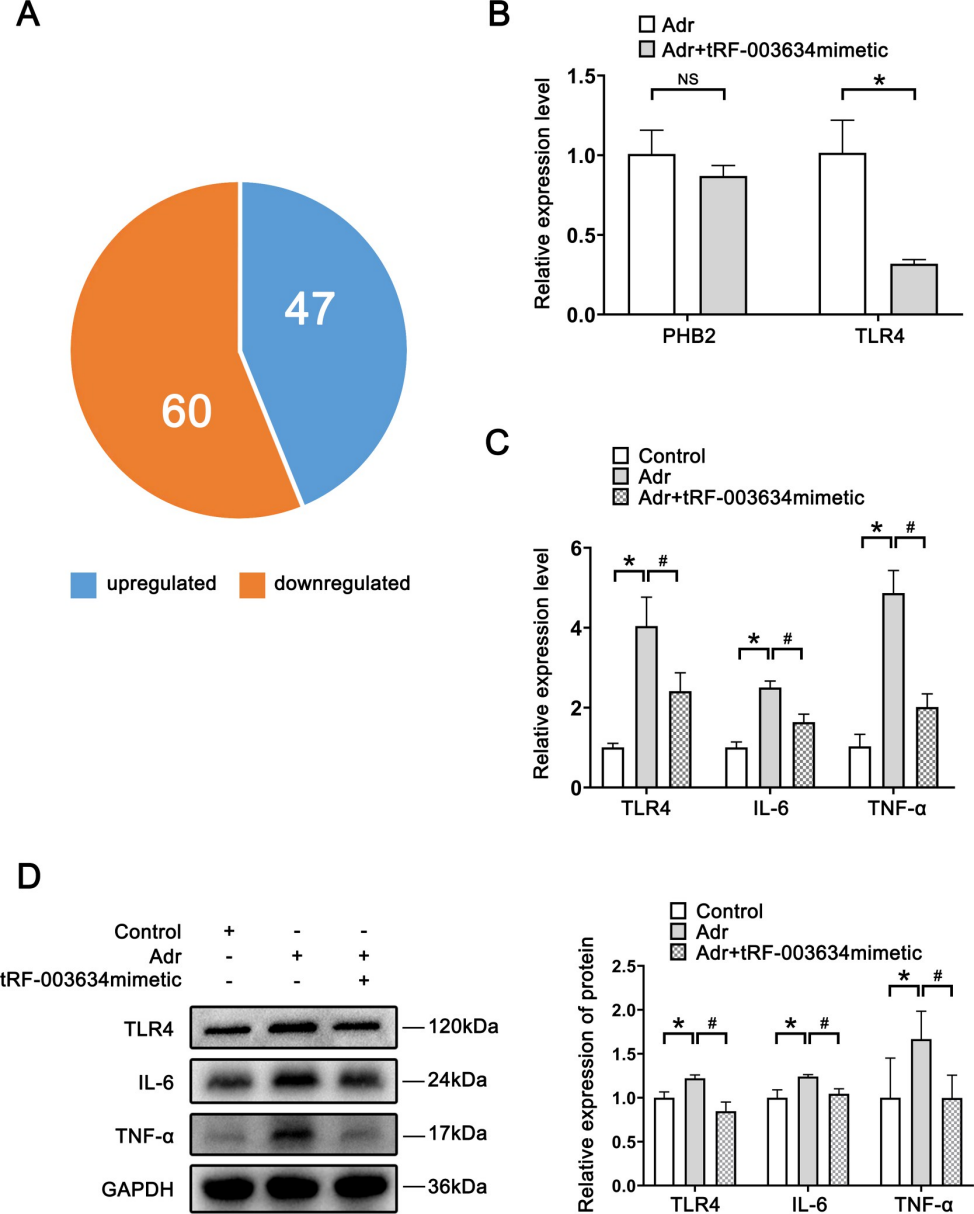
TLR4 is the downstream target gene regulated by tRF-003634. (A) Gene differential expression after tRF-003634 overexpression. (B) Expression of PHB2 and TLR4 were analyzed by qRT-PCR. (C) Expression levels of TLR4, IL-6 and TNF-α in podocytes were demonstrated by qRT-PCR. (D) Western blot analysis of expression levels of TLR4, IL-6 and TNF-α in podocytes. n = 3, *P<0.05 vs. the control group; #P<0.05 vs. the Adr group. NS, non-significant.

High-throughput mRNA sequencing suggested that compared with the Adr group, 107 mRNA expression levels were significantly different in the tRF-003634 mimetic group (multiple of difference greater than two-fold, P<0.05), among which 47 were upregulated and 60 were downregulated (Fig 3A).

The target genes prohibitin 2 gene (PHB2) and TLR4, having large differences between the two groups and low expression in the Adr+tRF-003634 mimetic group, were selected. They have been reported to be associated with podocyte injury. The mRNA expression levels of PHB2 and TLR4 were detected by qRT-PCR. Compared to the Adr group, TLR4 mRNA expression in the Adr+tRF-003634 mimetic group was found to be significantly decreased (P<0.05), while PHB2 mRNA expression was not significantly different, indicating that TLR4 may be a downstream target gene regulated by tRF-003634 (Fig 3B).

The expression levels of TLR4, IL-6, and TNF-α were detected by qRT-PCR and western blot; the expression levels of TLR4, IL-6, and TNF-α in the Adr group were significantly higher than that in the control group (P<0.05). The expression levels of TLR4, IL-6, and TNF-α in the Adr+tRF-003634 mimetic group were significantly lower than those in the Adr group (P<0.05; Fig 3C and 3D). Thus, these findings suggest that tRF-003634 reduces Adr-induced podocyte injury by reducing the expression of TLR4 and downregulating the secretion of proinflammatory cytokines by podocytes.

### tRF-003634 alleviated Adr-induced podocyte injury by reducing the stability of TLR4 mRNA through YTHDC1

**Fig 4:**
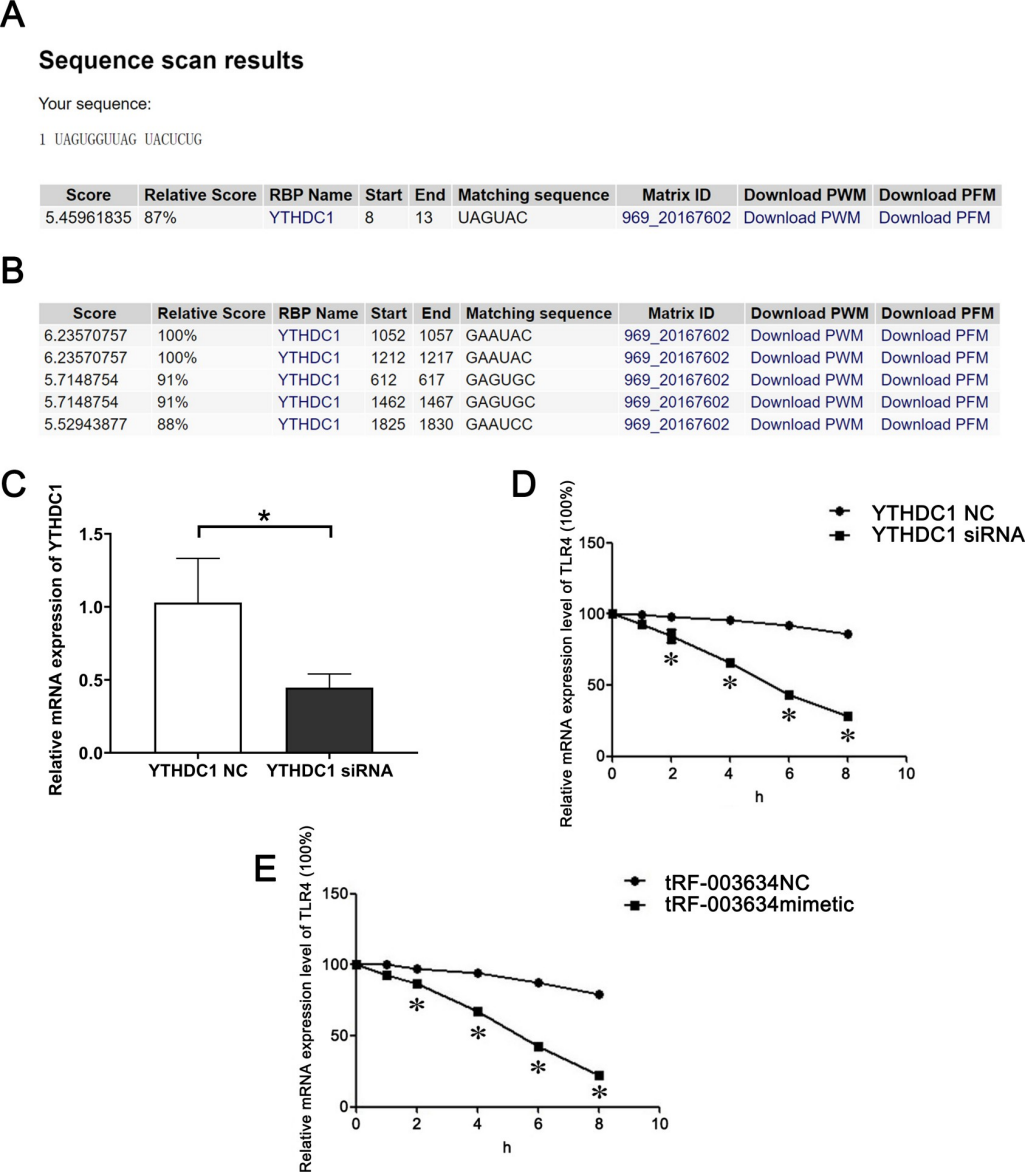
tRF-003634 alleviates Adr-induced podocyte injury by reducing the stability of TLR4 mRNA through YTHDC1. (A) The binding of tRF-003634 to YTHDC1. (B) The binding of TLR4 to YTHDC1. (C) The interference efficiency of YTHDC1 was determined using the qRT-PCR. (D) Expression levels of TLR4 mRNA were detected by qRT-PCR after the knockdown of YTHDC1. (E) Expression levels of TLR4 mRNA were evaluated by qRT-PCR after the overexpression of tRF-003634. n = 3, *P<0.05 vs. respective NC. YTHDC1 NC, YTHDC1 siRNA negative control; tRF-003634 NC, tRF-003634 negative control.

To further discuss the mechanism by which tRF-003634 regulates TLR4 expression, we performed a bioinformatic analysis using the RBP prediction website: YTH domain-containing protein 1 (YTHDC1) may be the common RBP of tRF-003634 and TLR4, and the possible binding site is "UAGUAC" (Fig 4A and 4B).

To determine whether YTHDC1 is related to TLR4 mRNA stability, we knocked down YTHDC1 in podocytes using siRNA technology. The interference efficiency was verified using qRT-PCR (Fig 4C). qRT-PCR analysis revealed that TLR4 mRNA stability decreased after YTHDC1 knockdown compared with the YTHDC1 NC group (Fig 4D). TLR4 mRNA stability was examined after the overexpression of tRF-003634 in podocytes; qRT-PCR analysis showed that the stability of TLR4 mRNA in the tRF-003634 mimetic group was lower than that in the tRF-003634 NC group (Fig 4E). These results prompted that the mechanism by which tRF-003634 regulates TLR4 expression may be related to YTHDC1.

## Discussion

CKD is a major public health concern that threatens human health. However, few drugs can be clinically used to treat it, and there is currently no cure. In-depth exploration of the pathophysiological mechanisms of CKD development and new therapeutic strategies are urgently required. Podocytes are key cells that affect the occurrence and development of CKD [5]. Podocytes cover the outer layer of the GBM and constitute an essential part of the glomerular filtration barrier. Disruption of the GBM is a main cause of massive proteinuria, and many mutations resulting in nephrotic syndrome are closely related to the encoding of podocyte-specific proteins [7]. Acquired podocyte dysfunction occurs in nearly all forms of kidney disease [16]. Podocyte injury is reported to play a crucial role in the development of proteinuria, and is closely correlated with the progression of CKD in humans and experimental animal models [4, 17]. Decoding the mechanism of podocyte injury may provide more avenues for preventing and treating CKD.

Abnormal expression of tRFs is linked to various tumors, including breast cancer, and to the role of oncogenes or tumor suppressor genes [18]. Compared with miRNAs and lncRNAs, tRFs have more diverse mechanisms of action, and play important regulatory roles in multiple biological processes, such as tumor cell proliferation, apoptosis, invasion, metastasis, and drug resistance [8, 19]. This study used high-throughput sequencing to screen differentially expressed tRFs during Adr-induced podocyte injury. Subsequently, tRF-003634, which was predicted to be associated with podocyte injury, was selected for further research. It was predicted to be expressed at low levels in the Adr-treated group; gene ontology and kyoto encyclopedia of genes and genomes pathway analyses suggested that it may be involved in podocyte injury [14]. qRT-PCR demonstrated that the expression level of tRF-003634 in the Adr group was significantly lower than that in the NC group, which was consistent with the sequencing results. tRF-003634 was significantly downregulated in Adr-induced podocyte injury, suggesting that it may be involved.

To explore the potential effects of tRF-003634 in podocyte injury, we used Adr to make a model in cell and animal experiments. Changes in nephrin and podocin levels were examined by qRT-PCR and western blot to observe podocyte damage. In vitro and in vivo, the expression levels of nephrin and podocin were significantly decreased in the Adr group, whereas their expression in the tRF-003634 group was significantly increased compared with that in the Adr group. Meanwhile, in the Adr nephropathy mouse model, urinary protein quantification and renal pathological changes in the tRF-003634 treatment group were alleviated compared with those in the Adr group, revealing that overexpression of tRF-003634 can alleviate Adr-induced podocyte injury. Therefore, we speculate that tRF-003634 is not only downregulated in podocyte injury but also has important biological functions. Further research on the role and mechanism of tRF-003634 in the process of podocyte injury is required.

To further investigate the mechanism by which tRF-003634 regulates podocyte injury, potential target genes of tRF-003634 were screened using high-throughput mRNA sequencing. TLR4 was selected for verification. Compared to the Adr group, the expression of TLR4 mRNA in the tRF-003634 group was significantly decreased, suggesting that TLR4 may be a downstream target gene regulated by tRF-003634.

Toll-like receptors (TLRs) bridge innate and adaptive immunity. Thirteen mouse TLRs have been identified [20, 21], and TLR4 was the first pattern recognition receptor to be discovered. TLR4 is a type I transmembrane protein and consists of an extracellular leucine-rich repeat structure, an intracellular conserved receptor domain, and a transmembrane domain. TLR4 is not only expressed in B cells, T cells, and macrophages but also in renal cells, such as podocytes, renal tubular epithelial cells, and mesangial cells [22–24]. TLR4 expressed by podocytes is involved in the local inflammatory response in the glomerulus; the upregulation of TLR4 promotes the secretion of proinflammatory cytokines, including IL-1, IL-6, TNF-α, and MCP-1 by podocytes, which ultimately leads to glomerular injury [25]. In addition, the TLR4 signaling pathway is activated in the inflammatory response of various kidney diseases, such as acute ischemic kidney injury, diabetic nephropathy, and obesity-related nephropathy [23, 24, 26]. In this study, after Adr-induced podocyte injury, the expression of TLR4 was significantly increased, and the expression of the inflammatory cytokines TNF-α and IL-6 was significantly increased. In contrast, overexpression of tRF-003634 significantly decreased the expression of TLR4, TNF-α, and IL-6. Therefore, tRF-003634 may reduce Adr-induced podocyte injury by reducing the expression of TLR4 and downregulating the secretion of pro-inflammatory cytokines by podocytes. However, the mechanism by which tRF-003634 regulates TLR4 expression is not clear, and we further investigated it.

At present, the functional mechanism of tRFs is usually explored using methods similar to those used for miRNA research [27, 28]. Studies have confirmed that the mechanisms of action of tRFs are more diverse than those of miRNAs and lncRNAs [18]. tRFs regulate the expression of protein-coding genes at the post-transcriptional or translational level by binding to mRNA 3′-UTR, 5′-UTR, or RBP. In order to narrow down the scope of finding the mechanism of tRF-003634, we found that tRF-003634 (tRF-17-V47PU9p) was derived from tRNA His by querying the MINTBASE database. It is formed by cleavage of ANG at the 29th base of tRNA His and belongs to the i-tRF type. One of the main modes of action of i-tRF is to compete with target gene mRNA for binding to RBP, thereby affecting the stability of the target gene mRNA [29]. Therefore, the mechanism of action of tRF-003634 in reducing podocyte injury conforms to this rule.

Through bioinformatic analysis, using the RBP prediction website, it was found that TLR4 mRNA and tRF-003634 have the same sequence "UAGUAC,” and they may bind to YTHDC1 through this sequence. YTHDC1 is a YTH domain-containing protein with m6A-dependent RNA-binding activity, and participates in various RNA processes, such as mRNA splicing, nuclear export, decay in translation, and post-transcriptional regulation [30, 31]. Proteins containing YTH domains play an important role in post-transcriptional modification by regulating the expression of genes involved in cancer and other processes, including cell cycle progression, cell proliferation, migration and invasion, inflammation, immunity, and autophagy. YTHDC1 has alternative pre-mRNA splicing ability, and is closely related to cancer [32–34]. To clarify whether YTHDC1 is related to the mRNA stability of TLR4, we knocked down YTHDC1 in podocytes using siRNA technology. After verifying the interference efficiency, actinomycin D was used to inhibit gene transcription in the podocytes. Total RNA was collected at different time points (0, 1, 2, 4, 6, and 8 h) and the half-life of TLR4 mRNA was detected by qRT-PCR. Compared with the control group, the half-life of TLR4 mRNA was shortened, and the stability of TLR4 mRNA was decreased after the knockdown of YTHDC1, suggesting that YTHDC1 is positively correlated with the stability of TLR4 mRNA. Simultaneously, the stability of TLR4 mRNA was detected after tRF-003634 was overexpressed in podocytes; the half-life of TLR4 mRNA was shortened and its stability decreased after overexpression of tRF-003634. These results indicated that the mechanism by which tRF-003634 regulates TLR4 expression may be related to YTHDC1.

In conclusion, this study explored the role of tRF-003634 in Adr-induced podocyte injury and its possible mechanism in vitro and in vivo. The expression of tRF-003634 in Adr-induced podocyte injury was remarkably lower than in the control group. Overexpression of tRF-003634 alleviated Adr-induced podocyte injury in vitro and in vivo by reducing the stability of TLR4 mRNA. However, the mechanism by which tRF-003634 downregulates TLR4 mRNA expression remains unclear. We speculate that tRF-003634 can bind to YTHDC1 by competing with TLR4 mRNA, thereby reducing TLR4 mRNA stability, downregulating TLR4 expression, and ultimately protecting podocytes from injury. For further research, we will explore the roles of TLR4 and YTHDC1 in more depth. The elucidation of the mechanism of tRF-003634 alleviating podocyte injury can provide new strategies for CKD prevention and treatment.

## Supporting information

S1 Raw images (PPTX)
