# Supplementary figures and images for: tRF-003634 alleviates adriamycin-induced podocyte injury by reducing the stability of TLR4 mRNA
Source: PLoS One. 2023 Oct 19;18(10):e0293043. doi: 10.1371/journal.pone.0293043 (PMC10586663; doi:10.1371/journal.pone.0293043)

## Slide 1
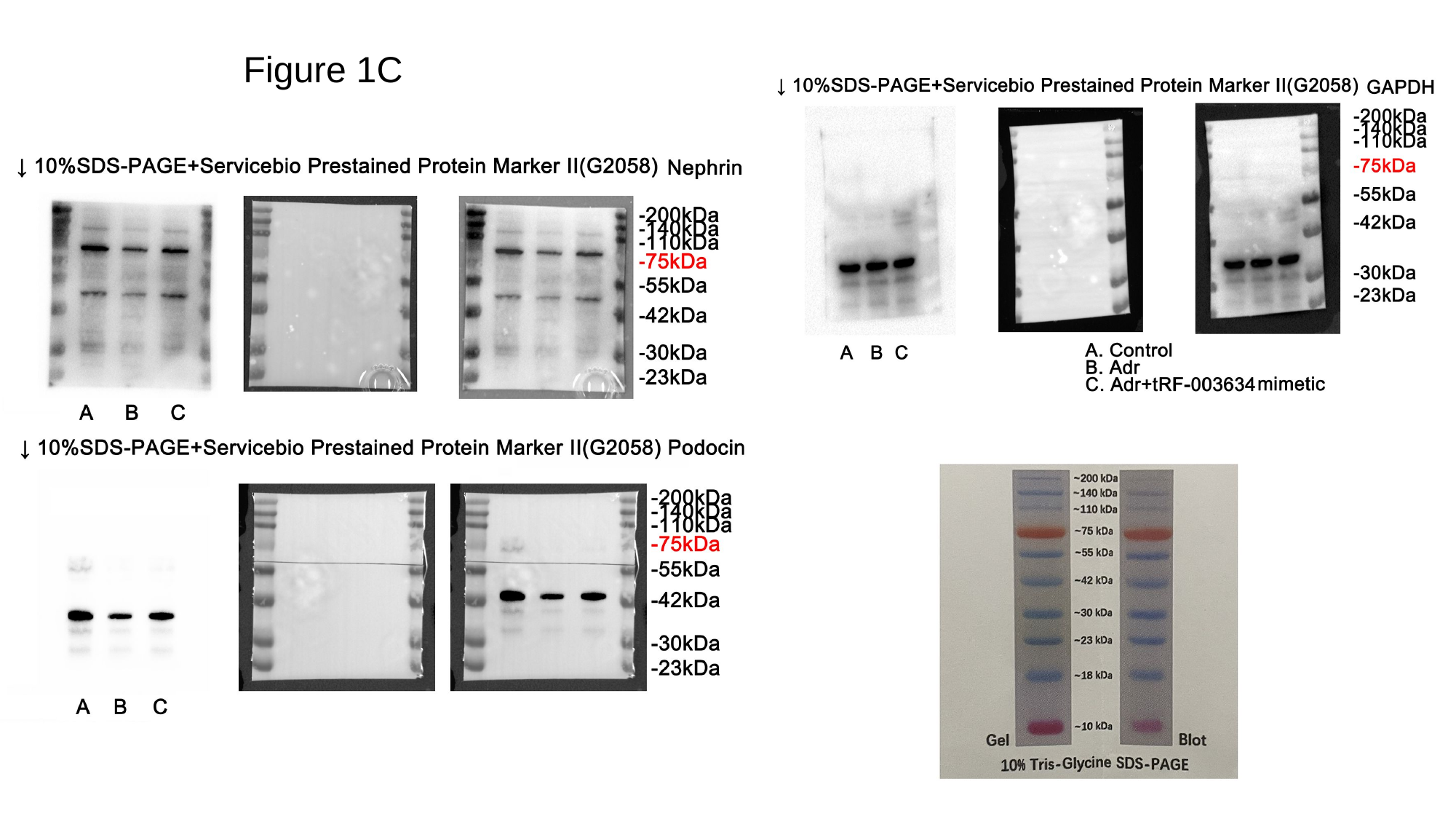

Figure 1C

## Slide 2
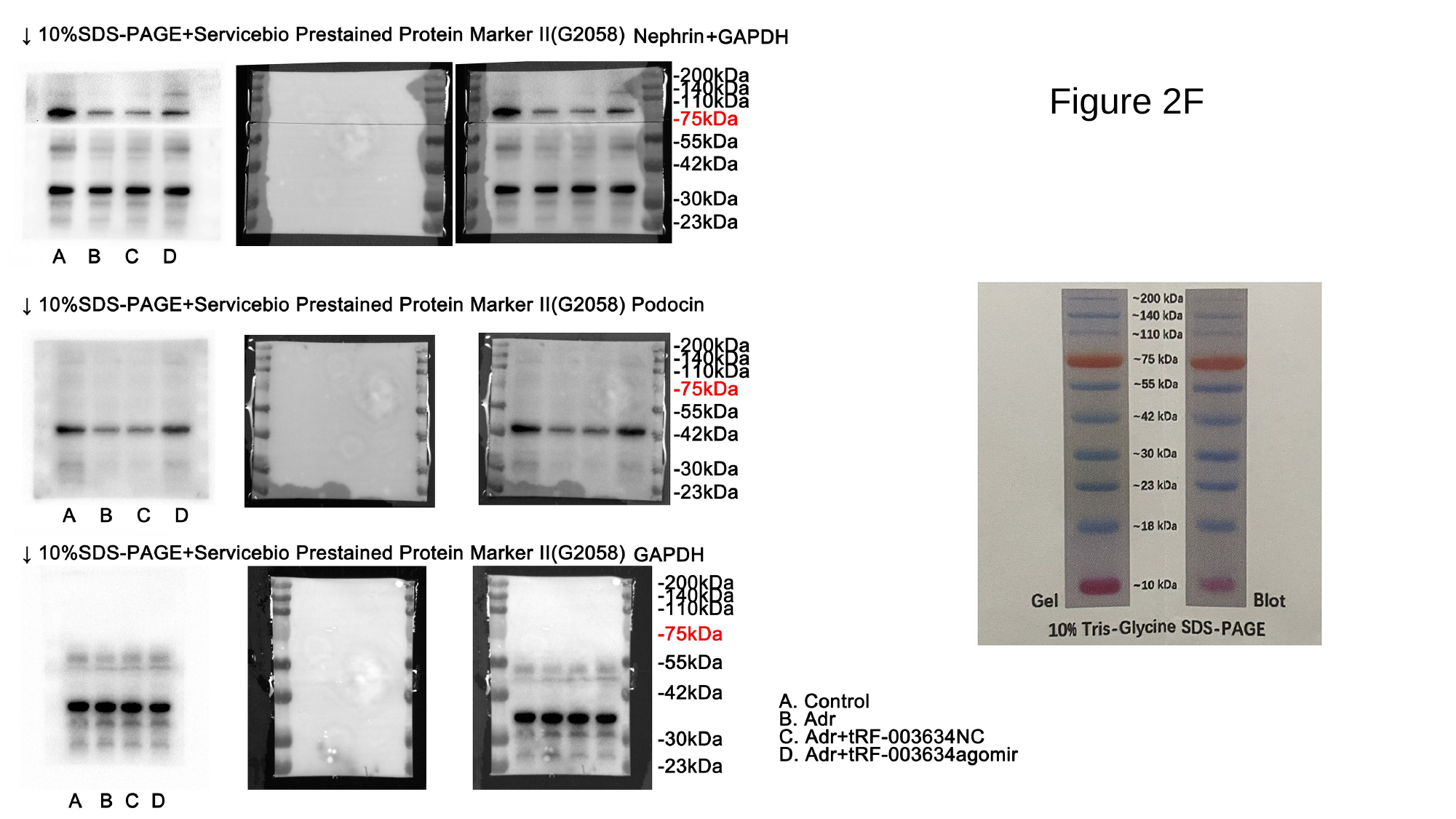

Figure 2F

## Slide 3
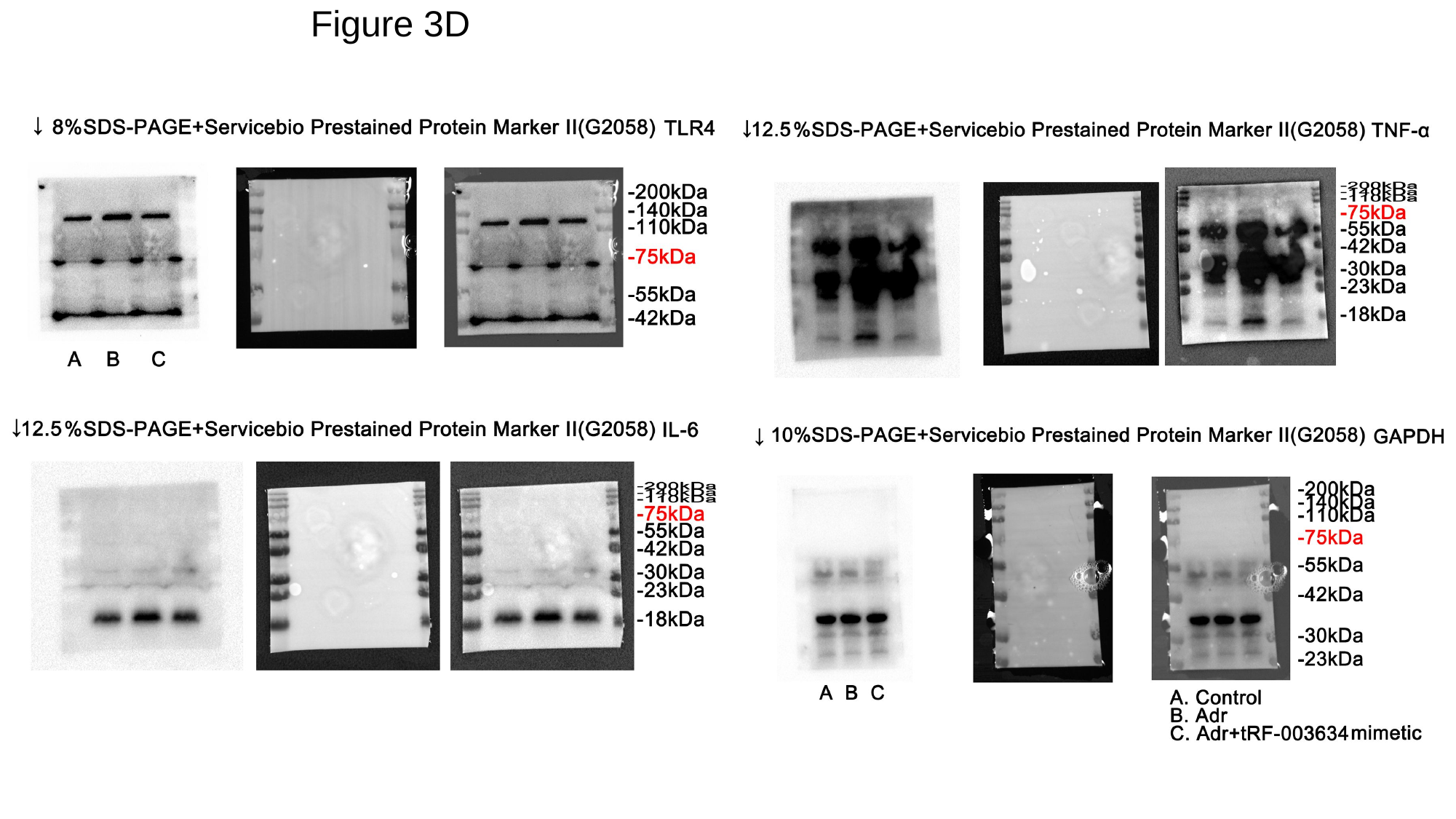

Figure 3D

Supplement: S1 Raw images — (PPTX) [file pone.0293043.s001.pptx]
